# Magnetic Resonance Assessment of Ejection Fraction Versus Echocardiography for Cardioverter-Defibrillator Implantation Eligibility

**DOI:** 10.3390/biology10111108

**Published:** 2021-10-27

**Authors:** Călin Schiau, Daniel-Corneliu Leucuța, Sorin Marian Dudea, Simona Manole

**Affiliations:** 1 Department of Radiology, “Iuliu Hatieganu” University of Medicine and Pharmacy, 400006 Cluj-Napoca, Romania; schiau.calin@umfcluj.ro (C.S.); sdudea1@gmail.com (S.M.D.); simona.manole@gmail.com (S.M.); 2 Department of Medical Informatics and Biostatistics, “Iuliu Hatieganu” University of Medicine and Pharmacy, 400349 Cluj-Napoca, Romania

**Keywords:** cardiovascular magnetic resonance, echocardiography, ejection fraction, implantable cardioverter defibrillator, nonischemic cardiomyopathy

## Abstract

**Simple Summary:**

Nonischemic cardiomyopathies with low left ventricular ejection fractions (LVEF) are eligible for an implantable cardioverter defibrillator. However, the guidelines do not specify which method should be used to assess LVEF. In our study we investigated the potential impact of performing two-dimensional echocardiography (2DE) compared to cardiovascular magnetic resonance (CMR) for LVEF regarding ICD eligibility. We found that 2DE both overestimated and especially underestimated the need for implantation, which can have serious implications in the quality of life and the prevention of death events.

**Abstract:**

Background: The aim of this study was to investigate the potential impact of performing two-dimensional echocardiography (2DE) compared to cardiovascular magnetic resonance (CMR) for left ventricular ejection fraction (LVEF) on implantable cardioverter defibrillator (ICD) eligibility. Methods: A prospective cohort of 166 consecutive patients with nonischemic cardiomyopathy (NICM) was designed to compare transthoracic 2DE and CMR imaging. Results: Echocardiography measurements have important differences and large limits of agreement compared to CMR, especially when assessing ventricle volumes, and smaller but relevant differences when assessing LVEF. The agreement between CMR and 2DE regarding the identification of subjects with EF <= 35, respectively <= 30, and thus eligible for an ICD measured by Cohen’s Kappa was 0.78 (95% CI: 0.68–0.88), *p* < 0.001, respectively 0.65 (95% CI: 0.52–0.78), *p* < 0.001. The disagreement represented 7.9%/11.3% of the subjects who had EF < 35%/< 30% as observed by CMR, who would have been classified as eligible for an ICD, resulting in an additional need to use an ICD. Moreover, 2.6%/3.3% would have been deemed eligible by echocardiography for an ICD. Conclusions: These measurement problems result in incorrect assignments of eligibility that may have serious implications on the quality of life and the prevention of death events for patients assessed for eligibility of an ICD.

## 1. Introduction

Cardiovascular diseases represent a main cause of death worldwide, and among European countries, Romania has a high cardiovascular disease (CVD) prevalence and mortality burden [1]. Cardiomyopathies are a heterogeneous group of myocardial diseases associated with mechanical and/or electrical dysfunction, accompanied or not by hypertrophy or dilation, of acquired or congenital causes. Cardiomyopathies affect up to 50% of patients with sudden cardiac death (SCD) in childhood or adolescence and a significant number of the candidates for a heart transplant [2]. Nonischemic cardiomyopathy is a subgroup of cardiomyopathies without any proven coronary artery disease, hypertension, valvular heart disease (VHD), or congenital heart disease [3].

Progression to heart failure (HF) and an increased risk of SCD are common aspects of cardiomyopathies. Studies found that the mortality decreased considerably after the implantation of an implantable cardioverter defibrillator (ICD) [4,5]. 

Current practice guidelines for the introduction of an ICD, in the primary prevention of SCD, state that left ventricular ejection fraction (LVEF) should be either <30% or 30–35%, depending on etiology and symptoms, but the technique by which it should be assessed is not specified [6,7]. The assessment of cardiac volumes and LVEF has valuable diagnostic, prognostic, and therapeutic implications for patients suffering from cardiomyopathy. Echocardiography has been widely used because it is readily available and noninvasive. LVEF measured by echocardiography is the best-validated among the predictors of SCD and is the primary parameter used in clinical practice to make treatment decisions [8]. However, there are studies that claim that cardiovascular magnetic resonance (CMR) has increased accuracy and reproducibility in LVEF assessment, particularly when compared with echocardiography [9,10,11].

Nevertheless, the agreement between the two diagnostic methods, especially their utility in ICD initiation needs to be further explored.

Therefore, the objectives of this study were the assessment of the agreement between two dimensional echocardiography (2DE) and CMR performed in patients with nonischemic cardiomyopathy (NICM) regarding the assessment of LVEF, end-diastolic volume (EDV) and end-systolic volume (ESV), followed by checking the agreement of the two methods to initiate an ICD based on the classical LVEF cutoffs.

## 2. Materials and Methods

### 2.1. Study Design and Setting

A prospective cohort study on consecutive patients was performed, evaluated between August 2016 and August 2019. The study cohort (*n* = 166) consisted of clinical patients who underwent 2DE and CMR examinations during hospitalization in a tertiary regional hospital from Romania. Written informed consent was obtained for all subjects included in the study. The University Ethics Commission approved the study protocol.

The inclusion criteria were: patients with NICM, cardiac volumes and LVEF measured by both CMR and echocardiography methods during their hospitalization, with no more than 5 days (usually 3 days) between the two investigations.

The exclusion criteria were:

- related to echocardiography (comorbidities altering the quality of echocardiography):
  - obesity;
  - emphysema;
- specific to CMR:
  - claustrophobia;
  - metallic prosthetic implants;
  - known allergy to the contrast agents;
- related to patient conditions:
  - uncontrolled arrhythmias;
  - pregnancy or lactation;
  - high-grade valve diseases or shunts;
  - a cardiac resynchronization or a cardiac revascularization procedure that happened between 2DE and CMR examinations.

Patients underwent echography and CMR examinations. The examiners had access to clinical and laboratory results of the patients but did not have access to the other examination (blind assessment).

### 2.2. Echocardiography

All 2DE images were acquired on the same device—a Vivid S70 scanner (GE Healthcare, Horten, Norway) using a 2D matrix array transducer (M5S). The images were stored in Digital Imaging format (EchoPac BT13, GE Vingmed Ultrasound). All stored images were obtained in the apical four- and two-chamber, respectively, standard parasternal long- and short-axis views. All cine-loops were analyzed by different experienced senior physicians unaware of the CMR results.

After manual tracing of the endocardial limits from the four- and two-chamber images, left ventricle (LV) volumes and LVEF were calculated using the Simpson’s biplane method, by different senior physicians that were involved in providing the health care for their own patients. As in the CMR examination, trabeculations and papillary muscles were included in the LV volumes.

Volume assessment was based on tracings of the blood–tissue interface in the apical four- and two-chamber views [8].

Global LV function was assessed by measuring the difference between the EDV and ESV divided by EDV. End diastole was considered the image from the cardiac cycle in which the respective LV dimension or volume measurement was the largest, as in the CMR examination, which is usually at the beginning of the R wave. End systole was considered the image after aortic valve closure, or the image in which the dimension or volume of LV was the smallest [8]. Avoiding ectopic and postectopic beats, EDV and ESV values from three cardiac cycles were obtained, and averaged. The LVEF values measured by 2DE were extracted from the clinical reports.

### 2.3. Cardiovascular Magnetic Resonance—Acquisition and Analysis

All subjects underwent CMR scans on the same device—a 1.5-T Avanto MRI scanner system (Siemens Medical Systems, Erlangen, Germany). An eight-element phased-array surface coil device was used. Retrospective electrocardiographic (ECG) gating was used, a breath-hold of 10 to 15 s for LV volumes and LVEF assessment being needed.

LVEF can be calculated when using CMR by various techniques: automated, semiautomated or manual methods [12]. The most commonly used is the Simpson disk summation method using short-axis cine steady-state free precession images (SSFP) of the LV—this method was used in the present study [13].

A stack of SSFP images were acquired in the short-axis plane from the level of the mitral valve annulus to the LV apex, with the following parameters: slice thickness 8 mm no gap; average in-plane resolution 1.3 × 1.3 mm^2^; TR 3.8 ms; TE 3.3 ms; 30 phases; flip angle 70°. A stack of SSFP images in three long-axis planes were also obtained—two-, three-, and four-chamber views.

A phase-sensitive inversion recovery sequence (PSIR) for late gadolinium enhancement (LGE) was performed 10 min after the intravenous administration of 0.1 mmol/kg of Gadobutrol (Gadovist, Gadavist; Bayer Pharma AG, Leverkusen, Germany). A stack of left ventricle, diastolic, short-axis images was obtained, with the following settings: field of view 360 × 290 mm^2^; voxel size 1.9 × 1.4 × 8 mm^3^; TE 3.17 msec; TR 1× RR interval; flip angle 25°.

Before the LGE images were obtained, an inversion time (TI) value for an optimal nulling of the normal myocardium was determined. This has been assessed on a midventricular short-axis slice, using a scout with increasing TI values.

LV volumes and LVEF measurements were based on successive short-axis stacks of cine images using QMASS software (Medis Medical Imaging, Leiden, The Netherlands). The CMR images were acquired by a senior reader. LVEF was measured by manual planimetry of the LV endocardium in short-axis cine images at ES and ED. ES and ED phases were chosen independently by each observer. Each of them took into account the maximum and minimum volume, with several phases contoured in case of doubt. At the basal slice, the LV cavity was differentiated from the atrium by the presence of ventricular myocardium, which was subsequently verified on a co-registered long-axis image. Papillary muscles were not excluded from the LV chamber tracing. LV volumes and mass were normalized to body surface area (BSA).

### 2.4. Statistical Analysis

Qualitative measurements were shown as absolute and relative frequencies. Normally distributed data were presented as means and standard deviations. Non-normally distributed data were presented as medians and quartiles. Using the Bland–Altman plot [14], the agreement between the two methods measured quantitatively was assessed.

The limits of agreement were computed classically, using the 1.96 * standard deviation of the difference between the methods, as well as using the nonparametric Harrell Davis quantile estimator [15], along with bias-corrected, accelerated bootstrap 95% confidence intervals [16].

Agreement between the two methods for different thresholds of ejection fraction classification was assessed with Cohen’s Kappa, bias-adjusted Kappa, and the prevalence-and-bias-adjusted Kappa, along with the *p*-value. When assessing different classification possibilities using LVEF in the view of a future eligibility for an ICD, we considered CMR as the best method (the standard method) for measuring LVEF. A *p*-value beneath 0.05 was considered statistically significant, for all statistical tests.

The R environment for statistical computing and graphics, version 4.1.0 [17], was used for all statistical calculation.

## 3. Results

### 3.1. Baseline Patient Characteristics

The study sample consisted of 166 patients diagnosed with NICM such as dilated, hypertrophic, or myocarditis. All patients completed both echo and CMR studies. Eleven patients were excluded: three because of uncontrolled arrhythmias, six as a result of interrupted or inadequate magnetic resonance examinations, two because they had a cardiac resynchronization, or a cardiac revascularization procedure happened between 2DE and CMR examinations.

The mean age was 46.35 years (16.6—standard deviation), ranging from 9 to 81 (Table 1). The majority of the patients were males, 119 (71.69%).

### 3.2. Agreement between CMR and Echocardiography Measurements

**Figure 1:**
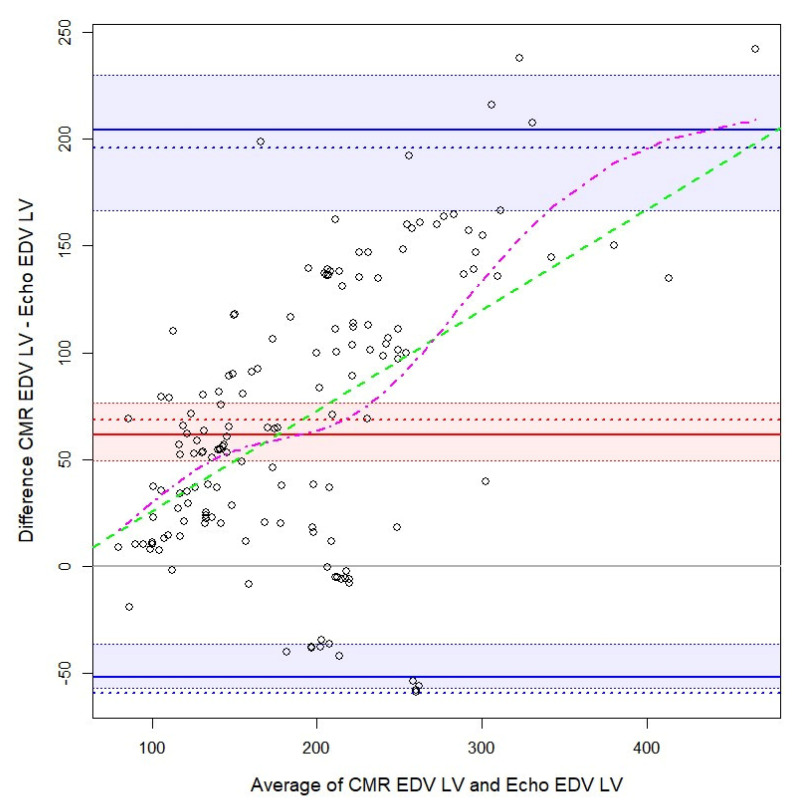
Bland–Altman plot for cardiac magnetic resonance imaging and cardiac echography of left ventricle end-diastolic volume.

**Figure 2:**
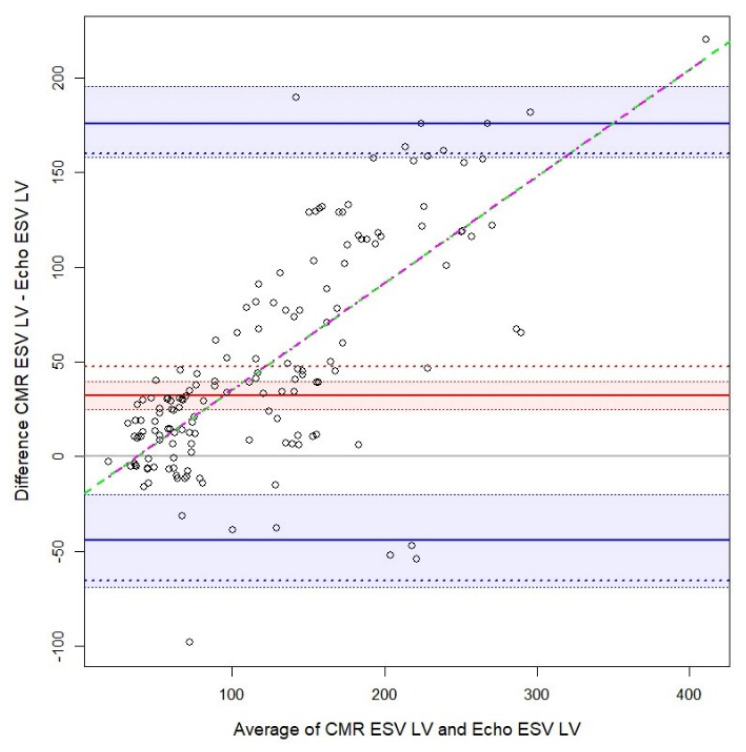
Bland-Altman plot for cardiac magnetic resonance imaging and cardiac echography of left ventricle end-systolic volume.

**Figure 3:**
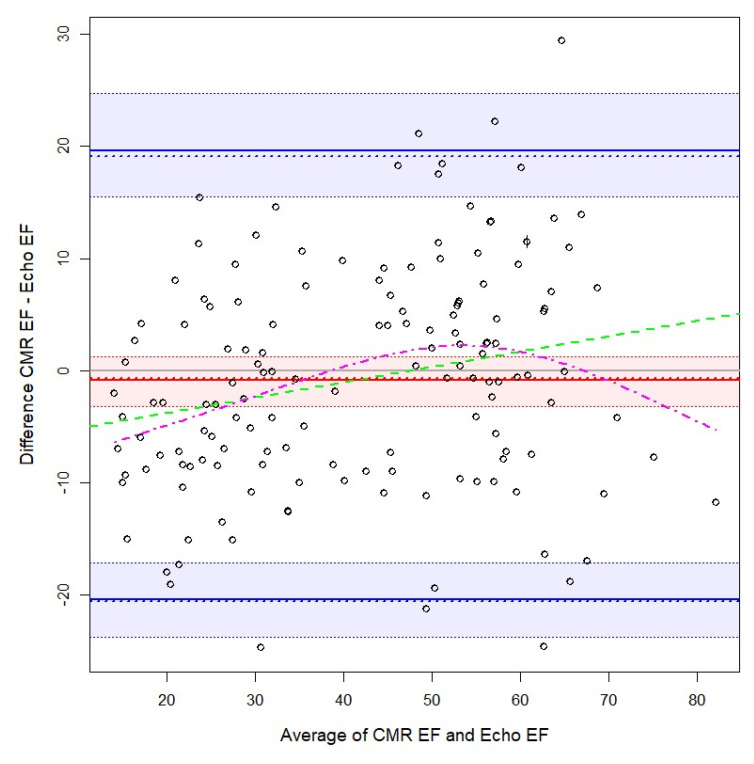
Bland-Altman plot for cardiac magnetic resonance imaging and cardiac echography of ejection fraction.

The agreement between CMR and echocardiography was evaluated for EDV LV, ESV LV, and EF (Table 2). Several measurements in the Bland–Altman plots (Figure 1, Figure 2 and Figure 3), indicated the non-normality of the difference or the average between the methods. Thus, Table 2 and the Bland–Altman plots show, besides the classical Bland–Altman statistics, the bias and limits of agreement computed using quantiles and bootstrapped confidence intervals. The highest bias (difference between the measurements of the two methods) was observed for EDV LV, about twice as large as for ESV LV, echography underestimating the measures by a median of 62.2 and 31.3. The limits of agreement are quite large, especially for EDV LV. Next, the bias was small for EF. Nevertheless, for EF the confidence interval for the bias had a 4.5% width. The limits of agreement were especially large for EF. For all the four measurements, the variability was not constant, instead, it increased—as the average between the two methods increased, the variability of the difference increased too. This was more evident for the EDV and ESV LV. Furthermore, there was a positive trend for EDV, ESV LV, showing that as the average between the two methods increased, the difference between the measures increased too. Moreover, we checked if the relation between the average and the difference between the methods was linear or not, and it was found that it is slightly nonlinear in the case of EDV LV, and EF.

CMR, cardiac magnetic resonance imaging, EDV, end-diastolic volume; LV, left ventricle; continuous red line, bias as 50th quartile; dotted red line, bias as mean; red interval, 95% bootstrapped confidence interval for 50th quartile; straight blue lines, limits of agreement at 2.5th and 97.5th quartiles; blue interval, 95% bootstrapped confidence interval for limits of agreement at 2.5th and 97.5th quartiles; dotted blue lines, limits of agreement at 1.96 standard deviations from the mean; dashed green line, linear regression line; magenta dot-dashed line, general additive model fit with a spline.

CMR, cardiac magnetic resonance imaging, ESV, end-systolic volume; LV, left ventricle; continuous red line, bias as 50th quartile; dotted red line, bias as mean; red interval, 95% bootstrapped confidence interval for 50th quartile; straight blue lines, limits of agreement at 2.5th and 97.5th quartiles; blue interval, 95% bootstrapped confidence interval for limits of agreement at 2.5th and 97.5th quartiles; dotted blue lines, limits of agreement at 1.96 standard deviations from the mean; dashed green line, linear regression line; magenta dot-dashed line, general additive model fit with a spline.

CMR, cardiac magnetic resonance imaging; EF, ejection fraction; LV, left ventricle; continuous red line, bias as 50th quartile; dotted red line, bias as mean; red interval, 95% bootstrapped confidence interval for 50th quartile; straight blue lines, limits of agreement at 2.5th and 97.5th quartiles; blue interval, 95% bootstrapped confidence interval for limits of agreement at 2.5th and 97.5th quartiles; dotted blue lines, limits of agreement at 1.96 standard deviations from the mean; dashed green line, linear regression line; magenta dot-dashed line, general additive model fit with a spline.

### 3.3. The Specific Value of EF Agreement between CMR and Cardiac Echography and Its Implication in ICD Eligibility

**Figure 4:**
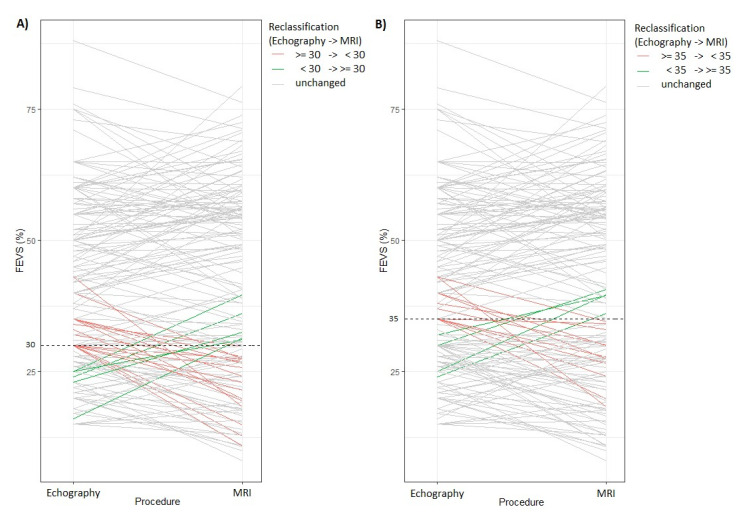
Reclassification of ejection fractions from echocardiography measurements to CMR measurements, for two thresholds used for implementing ICD therapy. (**A**) Patients reclassified for a 30% FEVS threshold, (**B**) Patients reclassified for a 35% FEVS threshold.

The observed agreement between CMR and echocardiography regarding the identification of subjects with EF <= 35, and thus eligible for an ICD are presented in Table 3 (Figure 4). The Cohen’s Kappa was 0.78 (95% CI: 0.68–0.88), *p* < 0.001, while the bias-adjusted Kappa, and the prevalence-and-bias-adjusted Kappa were 0.78 and 0.79, indicating a statistically substantial agreement. Nevertheless, clinically, 10.5% of the cases were in disagreement; 7.9% of the subjects who had EF < 35% as observed by CMR would have been classified as eligible for an ICD (having EF >= 35% as observed by echocardiography), resulting in an additional need to use an ICD, according to the guidelines. Moreover, 2.6% would have been deemed eligible by echocardiography, even if, according to CMR they would not be eligible, resulting in patients receiving ICD therapy although not necessary. A sensitivity analysis for subjects with CMR EF < 50% showed an increase to 13.8% for subjects who had EF < 35% as observed by CMR, and who would have been classified as eligible for an ICD, even if according to echography they did not need it, while 4.6% would have been deemed eligible by echocardiography, even if, according to CMR they would not be eligible.

When assessing agreement between CMR and echocardiography regarding the identification of subjects with EF >= 30, as a threshold for ICD eligibility, the Cohen’s Kappa was of 0.65 (95% CI: 0.52–0.78), *p* < 0.001, while the bias-adjusted Kappa, and the prevalence-and-bias-adjusted Kappa were 0.65 and 0.71, indicating a statistically substantial agreement. As in the other threshold, 11.3% of the subjects would have been considered eligible for an ICD using CMR, even if according to echography they did not need it, while 3.3% would have received an ICD according to echography, even if they would have not need it according to CMR (Figure 4). A sensitivity analysis for subjects with CMR EF < 35%, showed an increase to 27% for subjects who had EF < 30% as observed by CMR, who would have been classified as eligible for an ICD, even if according to echography they did not need it, while 4.8% would have been deemed eligible by echocardiography, even if, according to CMR they would not be eligible.

## 4. Discussion

In the present study, we managed to assess the agreement of 2DE and CMR performed in patients with NICM regarding the assessment of LVEF, EDV and ESV, and then we compared the agreement of the two methods for initiation of ICD based on the classical LVEF cutoffs.

### 4.1. Quantitative Measurements Agreement

Regarding Bland–Altman analysis, the main findings were as follows: there were important measurement differences, as observed in the bias, between CMR and echocardiography regarding EDV LV, ESV LV; there were smaller measurement differences regarding LVEF; the limits of agreement were large for EDV LV, ESV LV, and LVEF; the measurements differences had high heterogeneity for EDV LV and ESV LV, while for LVEF were homogeneous. These results imply that echocardiography measurements had important differences compared to CMR in assessing the left ventricle dimensions, even if it is the largest structure of the heart [18]. If we consider that CMR is a more accurate measurement technique, then the credibility of echocardiography measurements is questionable. Even if the bias was relatively small in absolute values for LVEF compared with the other measurements, clinically it can have a big impact on selecting, or not, a subject for ICD therapy. Dilated cardiomyopathies (DCM) are determined by genetic or acquired disorders, which can express as dilation or LV dysfunction mildly or not at all, or sometimes their expression can increase over time and thus at initial times could be overseen. These aspects, combined with the low accuracy of 2D ultrasound in measuring volumes as highlighted in this study, can lead to a delay in diagnosis with numerous medical and economic implications. So, Pinto et al., proposed another category of DCM entitled Hypokinetic nondilated cardiomyopathy (HNDC), based on LV or biventricular global systolic dysfunction without dilation (defined as LVEF < 45%), not explained by abnormal loading conditions or coronary artery disease, thus eliminating the strict need for a large left ventricular cavity [19]. The electric instability of the heart caused by the DCM can occur in any place of the heart, although some are considered as important sources of ventricular arrhythmias, such as the left ventricle summit, with a high level of difficulty for treatment [20,21]. Thus, not only can the modified dimensions of LV with low LVEF influence the need for and success of an ICD, but so can the anatomical and physiological modifications induced by DCMs.

### 4.2. Qualitative EF Measurements’ Agreement and ICD Eligibility Implications

To further dwell on this problem, we explored how these measurement issues of echocardiography compared to CMR would influence ICD eligibility. Thus, we checked the agreement of identifying subjects with EF at two thresholds used for ICD eligibility, namely 35% and 30%. Although the agreement was substantial (as per rule of thumb in the literature, that are known to be relative guidelines which have to be taken with a pinch of salt), having values between 0.6 and 0.8 (kappa = 0.78 and 0.65, respectively), the agreement was not perfect, which it is required to be for assessing the eligibility for an intervention that can increase the quality of life and also might be lifesaving [22]. This implies the two methods will offer different results in a number of cases. For these thresholds, 7.9% (*n* = 12), and respectively, 11.3% (*n* = 17) of the subjects would have not received implantation based on echocardiography findings even they should have according to CMR findings. Moreover, 2.6% (*n* = 2), and respectively, 3.3% (*n* = 5) of the subjects would have received ICD implantation based on echocardiography findings even if it was not the case based on CMR findings. These findings are worrisome since both overestimation of the need for implantation and especially the underestimation of the need for implantation, can have serious results, in addition to problems of cost and inconvenience.

### 4.3. Comparison with Other Studies

In the study conducted by Joshi et al., at the EF threshold of 35%, CMR reclassified 21% of patients referred for an EF or ICD assessment. At the EF threshold of 30%, CMR reclassified 9.6% of patients. Their findings are in the same direction as our study but with higher values [23] in the analysis of all patients and similar values in the sensitivity analysis on patients with CMR-LVEF below 50. Similar findings were observed by Rijnierse et al., where at the EF threshold of 35%, CMR reclassified 19% of patients that received an ICD implantation and had a CMR-LVEF below 35. Application of a cardiac CMR-LVEF cutoff of 30% offered comparable results with a 2DE-LVEF cutoff value of 35% [24]. The differences between studies are due to different inclusion criteria.

There are studies that have found a similar result to our study (Figure 3), that at lower LVEF values, the 2DE commonly overestimates CMR LVEF, and at higher LVEF, echocardiography may underestimate CMR values, which could influence ICD eligibility, especially in the intermediate LVEF range [25] due to the fact that LVEF thresholds used for primary prevention of ICD placement are fixed.

The present study found a smaller disagreement—and thus reclassification (10.5% and 14.6%)—compared to the findings observed by Joshi et al. [23], who identified a reclassification of 21% concerning device eligibility when CMR was used for LVEF evaluation. The reclassification was higher (41% of the patients) in patients with 2DE-LVEF within the range of 30–35%, who were allocated ICD implantation most frequently. Even a small overestimation of LVEF by 2DE, such as one between 3% to 5% compared with CMR, is a difference that can become decisive in the range of 30–35% [26].

### 4.4. Discussion about Future of Diagnostic Method Roles in ICD Eligibility

Since 2D echography incorrectly dictates eligibility compared to CMR, one way to continue to use it but with fewer errors might be to use a higher threshold for 2D, so that patients that need an ICD will not be incorrectly deemed as ineligible. However, several studies are arguing that the current guideline cutoff value of <= 35% should be lowered to <= 30 [23,27]. Thus, this approach seems not to be in the right direction.

3D echocardiography underestimates volumes in most patients because it cannot differentiate between compact myocardium and trabeculae. However, recently, a new generation of fully automated software based on an adaptive analytics algorithm has been conceived for left heart-chamber quantification. This novel technology appears feasible, fast, and reproducible. Furthermore, automated default border detection based on the compact myocardium with operator’s adjustments has shown an excellent agreement with cardiac CMR [28].

A study conducted by Gaibazzi et al. [29], showed the potential of a new ultrasound technique for detecting myocardial fibrosis (scar imaging echocardiography with ultrasound multipulse scheme—eSCAR), compared to CMR assessing late gadolinium enhancement (CMR-LGE). The eSCAR technique was able to identify the myocardial scar similarly to CMR-LGE, having very close results regarding the presence, coronary territory and segmental extension, except for apical regions where CMR has demonstrated superior accuracy. However, eSCAR has certain advantages, being an application of an existing technology, which is widely available.

In addition, as shown in another study conducted by Gaibazzi et al. [30], the presence and extent of myocardial scar assessed by eSCAR was independently associated with appropriate ICD shocks in patients previously diagnosed with myocardial infarction and reduced LVEF.

Thus, the future diagnostic method for ICD eligibility would be most probably debated between CMR and 3D echography, and not 2D echography.

However, CMR is gradually becoming more accessible and faster, and breath-hold acquisition sequences now require significantly less time. Furthermore, there are studies [31] suggesting that CMR can be useful due to the benefit of LGE, which indicates fibrosis secondary to various causes including dilated cardiomyopathy, valvular heart disease, chronic ischemia or hypertension.

### 4.5. Generalisability

This study is representative to the majority of hospitals that deal with various types of cardiomyopathies and that are not usually specialized centers of ICD therapy. In an ICD center, the percentage of reclassifications would be higher than those found in our study, since we had a fair number of subjects with normal EF.

### 4.6. Limitations

The present study was not designed to assess the real impact of CMR, but to explore its potential impact. Only randomized clinical trials can assess the long-term effect of using echocardiography or CMR for ICD eligibility. Nevertheless, this study signals a situation with far-reaching clinical implications for cardiomyopathy patients.

Some of the inherent limitations of transthoracic 2DE are image-plane positioning errors, foreshortening of the LV long axis, geometric assumptions, and cardiac translation [32].

The time between the two investigations was about 3 days, with a maximum of 5 days, which could explain some of the variability between the two measurements. Nevertheless, the time between the interventions was short, and there were no important treatments (coronary revascularization or cardiac resynchronization) that could have influenced the measured variables.

We did not assess intra and inter-observer variability for CMR-LVEF, but other studies have shown that the interclass correlation coefficient for this measurement are very high [23].

### 4.7. Study Strengths

First, in terms of the number of subjects, this article is distinguished by a larger cohort compared to other studies that have chosen to address this topic [23,24,26].

Second, the present study used a more appropriate method to assess the bias and limits of agreement in the Bland–Altman framework, compared to other studies in the literature, since it used nonparametric methods for these estimations [15,16].

Third, LVEF assessments by 2DE were evaluated by multiple readers (the physicians that were treating their patients) who were unaware of the study objectives, a setting much closer to the common clinical practice—a pragmatic approach. This is an important point, since the results of this study are more generalizable to the current practice in hospitals, where those who perform echocardiography have different experiences.

The present study provides further support for a more refined choice of ICD therapy in patients with NICM. The technique used to measure LVEF in patients being considered for ICD therapy can have paramount clinical importance in the treatment, quality of life, and outcomes of patients. The debate is still open between a growing number of candidates: CMR, 3D echography, new 2D echography techniques. Future trials can solve this dilemma, although they have to enroll large cohorts of patients in order to have enough power to observe significant differences. Still, the increase in accuracy in measuring EF is advisable due to the price associated with ICD implantation [33].

## 5. Conclusions

2D echocardiography measurements have important differences when compared to CMR, especially when assessing EDV LV and ESV LV, and smaller differences when assessing LVEF. The limits of agreement are clinically important for all the four measurements. The agreement between echocardiography and CMR in recommending an ICD based on commonly used thresholds of 35% and 30% is not perfect, resulting in both overestimation of the need for implantation and especially the underestimation of the need for implantation. These can have serious implications on the outcomes of patients that are assessed for eligibility for an intervention, with implications on the quality of life and the prevention of death events. The present study suggests that 2D echocardiography is not a suitable method for the assessment of LVEF for ICD eligibility. Of course, clinical trials would be warranted to further check which assessment method will lead to improved outcomes for patients.

## Figures and Tables

**Table 1:** Patients’ characteristics, along with cardiac assessment using 2D echography and cardiovascular magnetic resonance.

Characteristic	Number (%) (*n* = 166)
Age (years), mean (SD)	46.35 (16.16)
Males, *n* (%)	119/166 (71.69)
BMI (kg/m^2^), median (IQR)	25.72 (23.35–29.36)
BSA, median (IQR)	1.95 (1.82–2.08)
Heart disease, *n* (%)	
Dilatative cardiomyopathy	102/166 (61.45)
Hypertrophic cardiomyopathy	21/166 (12.65)
Myocarditis	34/166 (20.48)
Fibrosis	9/166 (5.42)
Clinical symptoms	121/152 (79.61)
NYHA class	1: 43/141 (30.5)
	2: 41/141 (29.08)
	3: 56/141 (39.72)
	4: 1/141 (0.71)
High blood pressure	43/148 (29.05)
Smoker, *n* (%)	67/156 (42.95)
Diabetes, *n* (%)	32/156 (20.51)
LVEF CMR, median (IQR)	40.6 (25.2–56.15)
LVEF echography, median (IQR)	42 (29.5–55)
EDV-LV CMR, median (IQR)	207.25 (157.48–278.75)
EDV-LV echography, median (IQR)	150 (108–201)
ESV-LV CMR, median (IQR)	127 (66.62–201.4)
ESV-LV echography, median (IQR)	86 (52–132)
IVS CMR, median (IQR)	10 (9–11)
IVS echography, median (IQR)	11 (9–12)

SD, standard deviation; IQR, interquartile range; BMI, body mass index; BSA, body surface area; NYHA, New York Heart Association; FEVS, left ventricular ejection fraction; CMR, cardiovascular magnetic resonance imaging; ESV, end-systolic volume; EDV, end-systolic volume; IVS, interventricular septum.

**Table 2:** Bias, limits of agreement and correlation between CMR and echography.

	Bias * (95% CI)	Limits of Agreement *, lb~ub (95% CI)	Bias BA (95% CI)	Limits of Agreement BA, lb~ub (95% CI)
EDV LV	61.7 (95% CI 49.6–76.5)	−51.8 (95% CI −57.2–−36.5)~204.2 (95% CI 166.6–230.1)	68.3 (58.2–78.4)	−59.4 (−69.5–−49.3)~196 (185.9–206.2)
ESV LV	32.3 (95% CI 24.7–39.6)	−44.3 (95% CI −69.2–−20)~176 (95% CI 157.9–195.7)	47.3 (38.4–56.3)	−65.3 (−74.2–−56.3)~159.9 (151–168.9)
EF	−0.9 (95% CI−3.2–1.3)	−20.4 (95% CI −23.7–−17.1)~19.6 (95% CI 15.5–24.7)	−0.7 (−2.3–0.9)	−20.5 (−22.2–−18.9)~19.1 (17.5–20.7)

EDV, end-diastolic volume; LV, left ventricle; ESV, end-systolic volume; EF, ejection fraction; CI, confidence interval; lb, lower bound of limits of agreement; ub, upper bound of limits of agreement; BA, computed using Bland-Altman method (for limits of agreement using 1.96 * standard deviation of the difference between the methods); *, bias and limits of agreement computed using quantiles, while the confidence interval for the limits of agreement was computed using bootstrap.

**Table 3:** ICD eligibility at EF 35% and 30% thresholds, as seen by CRM and by echocardiography, for all the subjects as well for subgroups where CMR EF < 50% or CMR EF < 35.

	CMR EF		
Echography EF (all subjects)	>=35% *n* (% of total)	<35% *n* (% of total)	Total
>=35%	84 (55.6)	12 (7.9)	96
<35%	4 (2.6)	51 (33.8)	55
Total	88	63	151 *
Echography EF (CMR EF < 50%)	>=35%	<35%	Total
>=35%	20 (23.0)	12 (13.8)	32
<35%	4 (4.6)	51 (58.6)	55
Total	24	63	87 *
Echography EF (all subjects)	>=30%	<30%	Total
>=30%	96 (63.6)	17 (11.3)	113
< 30%	5 (3.3)	33 (21.9)	38
Total	101	50	151 *
Echography EF (CMR EF < 35%)	>=30%	<30%	Total
>=30%	10 (15.9)	17 (27.0)	27
<30%	3 (4.8)	33 (52.4)	38
Total	13	50	151 *

*—15 patients had missing data on the echography; ICD, implantable cardioverter defibrillator; EF measurement; CMR, cardiac magnetic resonance imaging; EF, ejection fraction.

## Data Availability

Data available on request due to privacy restrictions.
